# Changes in the bacterial communities in chromium-contaminated soils

**DOI:** 10.3389/fvets.2022.1066048

**Published:** 2023-01-04

**Authors:** Yiran Zhu, Kaimin Song, Guodong Cheng, Huiling Xu, Xiaozhou Wang, Changxi Qi, Pu Zhang, Yongxia Liu, Jianzhu Liu

**Affiliations:** ^1^College of Veterinary Medicine, Shandong Agricultural University, Tai'an, Shandong, China; ^2^Research Center for Animal Disease Control Engineering, Shandong Agricultural University, Tai'an, Shandong, China; ^3^The Affiliated Tai'an City Central Hospital of Qingdao University, Tai'an, Shandong, China

**Keywords:** microbiome, chromium, microbial diversity and structure, soil enzymes, MiSeq high-throughput sequencing

## Abstract

**Introduction:**

Hexavalent chromium or Cr(VI) is essential to various industries, such as leather manufacturing and stainless steel production. Given that inevitable leakage from industries pollutes the soil and thereby affects the soil environment. Microbial communities could improve the quality of the soil. Abundant bacterial communities would significantly enhance the soil richness and resist external pressure, benefiting agriculture. But the pollution of heavy metal broke the balance and decrease the abundance of bacterial communities, which weak the self-adjust ability of soil. This study aimed to explore changes in the diversity of soil bacterial communities and to identify the influences of soil bacterial communities on enzymes in soil polluted by Cr(VI).

**Methods:**

The target soils were sampled quickly and aseptically. Their chromium content was detected through inductively coupled plasma-mass spectrometry, and bacterial microbiome communities were explored through MiSeq high-throughput sequencing. Then, the content of nitrite reductase and catalases were investigated through enzyme-linked immunosorbent assay (ELISA).

**Results:**

Chromium content in polluted soils was higher than that in the control soils at all depths. Sobs, Chao1, Ace, and Shannon diversity estimators in the control were higher, whereas Simpson's diversity estimators in the control soils were lower than those of contaminated samples at all depths. Contaminants affected the composition of the bacterial community. The soil microbial species were relatively single and inhomogeneous in the polluted soils. The bacterial phyla in polluted and controlled soils include *Proteobacteria, Actinobacteria, Chloroflexi*, and *Acidobacteria*, which differ markedly in abundance.

**Discussion:**

The results of these observations provide insights into the ecotoxicological effects of Cr(VI) exposure to soil microorganisms. To sum up these results are critical for evaluating the stabilized state of microbial community structures, contributing to the assessment of the potential risk of metal accumulation in soils.

## 1. Introduction

Heavy metals are naturally occurring elements, including non-essential elements, such as lead (Pb), cadmium (Cd), and mercury (Hg), and biologically necessary elements, such as copper (Cu), manganese (Mn), and zinc (Zn). Some heavy metals in soil are physiologically necessary for plants and animals, exerting indirect or direct effect on agricultural output and public health. To date, concern about heavy metal pollution of soil has grown throughout the world (1).

Heavy metal pollution is a serious problem because threatens the natural environment, especially greenhouse soils, and human health by generating toxicity and promoting bioaccumulation (2). Soil contaminations threaten consumer health through food chain accumulation (3, 4). Large quantities of heavy metals negatively impact soils by reducing their productivity, especially percentage of seed germination, and bacterial biomass (5, 6). Previous research employing fingerprinting techniques demonstrated that heavy metal contamination has immediate and long-term consequences on terrestrial microbial communities (7, 8). The variety and structure of microorganisms are considerably altered after heavy metal pollution (8–10). However, few studies have revealed interactions among microorganisms in heavy metal-contaminated soils, and the energy and nutrition cycling of soil microbes are unknown.

In heavy metal-polluted soil, exceeding threshold levels of heavy metals, such as Cd and Pb, may have a deleterious impact on the quantity and quality (function and diversity) of the microbiota. Specifically, the high accumulation of significant metals might decrease microorganism community diversity, reducing resistance and resilience to environmental stress. Cr(VI) is the seventh most abundant element and is abundant in the Earth's crust (11). It is one of the most highly carcinogenic and soluble elements that exist as oxyanions, such as CrO${}_{4}^{2-}$ and Cr_2_O${}_{7}^{2-}$ (12). Cr(VI) was widespread use in some industries, such as leather manufacturing and stainless steel production; inevitable leakage through industrial wastewater leads to soil pollution, which further affects the soil environment (13).

Soil microbial communities improve soil quality (14, 15). The diversity of soil microbial communities is related to soil properties (16). However, how soil bacterial communities respond to changes in heavy metals, especially Cr(VI), remains uncertain. Soil bacterial communities are crucial indicators for soil pollution and can be investigated through Illumina high-throughput sequencing. In this study, Cr(VI)-polluted soil samples were examined *via* high-throughput sequencing technology, and the responses of soil bacterial communities to Cr(VI) were determined. The richness and evenness of microbial composition were analyzed in uncontaminated and polluted soils collected from three depths.

In the current study, we used the Illumina MiSeq technique to analyze microbial communities in abandoned and heavy metal-contaminated soils. We postulated that interactions between microbes and heavy metal contamination in abandoned factory soils impact microbial communities and that these altered interactions may have enabled bacteria to adapt to heavy metals.

## 2. Materials and methods

### 2.1. Field description and sample collection

This study was conducted in an abandoned factory (42° 03′N, 123° 29′E) in Shenyang, Liaoning Province, China. This region typically has a north temperate continental monsoon climate, characterized by a dry and cold winter, mild and rainy summer, and strong winds in spring and autumn. Two land types (uncontaminated and polluted) and three depths (0–20, 20–40, and 40–60 cm) were used in exploring the effects of Cr(VI) on soil bacterial communities. Using the five-point sampling approach and soil samplers, we collected 18 soil samples (50 g each). The samples were separated into two portions after homogenization and then placed in the dark. One portion was stored at 4°C; and the other, at room temperature. Each land type had three replicated sites (Table 1).

**Table 1:** Attribute of samples collected in the abandoned factory.

**Groups**	**Location**	**Soil condition**
Control soils	N42°03′57.77″-N42°03′57.95″	Soil exhibited the normal traits
	E123°29′27.34″-E123°29′27.92″	
Chromium soils	N42°03′57.99″-N42°03′58.31″	Soil showed the distinct yellowish brown
	E123°29′28.80″-E123°29′29.38″	

### 2.2. Detection of chromium contents in soils

Microwave digestion and inductively coupled plasma-mass spectrometry (ICP-MS) were conducted. In brief, soils (0.2500 g, dried, filtered through a 0.15-mm nylon screen) were digested in a disintegration tank containing 11 mL of a 6:3:2 mixture of nitric, hydrochloric, and hydrofluoric acid. Then, the mixture was supplemented with ultrapure water and heated again to discharge acid. Finally, the solution was placed in a 50 mL volumetric flask, and the volume was determined. The chromium levels of the soils were analyzed through ICP-MS (Hewlett-Packard, HP-4500, Avondale, PA, USA) according to previous methods (17).

### 2.3. Soil DNA extraction and sequencing

Soil microbial genomic DNA was isolated using an E.Z.N.A. soil DNA kit (Omega Bio-tek, Norcross, GA, U.S.) according to the manufacturer's instructions. DNA extracts were checked through 1% (w/v) agarose gel electrophoresis and stored for PCR amplification. The different regions of the 16S rRNA were amplified using special primers (V3–V4 regions for forward primers containing the sequence 5′-ACTCCTACGGGAGGCAGCAG-3′ and reverse primers 5′-GGACTACHVGGGTWTCTAAT-3′). The PCR protocol was as follows: 3 min denaturation at 95°C for the initial amplification, followed by 27 cycles at 95°C for 30 s, annealing at 55°C for 30 s, extension at 72°C for 45 s, and a final step at 72°C for 10 min. PCR products were purified using the AxyPrep DNA gel extraction kit (Axygen Biosciences, USA) according to the manufacturer's instructions and were quantified using the QuantiFluor-ST sensitive fluorometer (Promega, USA). High-throughput sequencing was performed on an Illumina MiSeq sequencing platform (Illumina, San Diego, USA) according to standard protocols. The obtained raw sequences were filtered according to quality.

### 2.4. Catalase (CAT) and nitrite reductase (NR) levels of soils

The catalase (CAT) and nitrate reductase (NR) levels of the soils were determined using commercially available kits (Cat: ml076929 and ml076879, Mlbio, Shanghai, China). Other procedures were performed by strictly following the manufacturer's instructions.

### 2.5. Bacterial diversity and statistical analysis

Operational taxonomical units (OTUs) with 97% similarity were clustered using the algorithm UPARSE version 7.1 (http://drive5.com/uparse/), and chimeric sequences were identified and removed using UCHIME. Alpha indices were acquired *via* Mothur 1.30.2 (https://www.mothur.org/wiki/Download_mothur). Tables for each taxonomy and beta diversity distance calculation were analyzed with Quiime 1.9.1 (http://qiime.org/install/index.html). The data were analyzed on the online platform of Majorbio Cloud Platform (www.majorbio.com) (18–21). The results are presented as the mean ± standard deviation and were checked by one-way ANOVA or student's *t*-test (*t*-test) using Statistical Package for the Social Sciences (version 19.0; SPSS Inc., Chicago, IL, USA). *P* < 0.05 indicated statistical significance.

## 3. Results

### 3.1. Chromium contents in soils

The measured soil chromium contents varied substantially between the two land types (uncontaminated and polluted) and among the three depths (0–20, 20–40, and 40–60 cm). The concentrations of chromium increased in polluted soils at all depths compared with those in reference soils. Meanwhile, chromium content decreased with increasing depth (Table 2).

**Table 2:** The contents of Cr (mg/L) in different groups.

**Depth (cm)**	**Control soils**	**Chromium soils**
0–20	4.597 ± 0.015	162.403 ± 0.569*
20–40	3.185 ± 0.003	136.291 ± 0.473*
40–60	1.784 ± 0.008	102.573 ± 0.430*

Data of diverse depths between control and chromium soil groups were represented as mean ± SD. Values in the same row with “*” were significantly different (*p* < 0.05) in the same depth between control and chromium groups, respectively. All data in the experiment were performed for triple independent experiments.

### 3.2. Alteration to soil microbial diversity index

**Figure 1:**
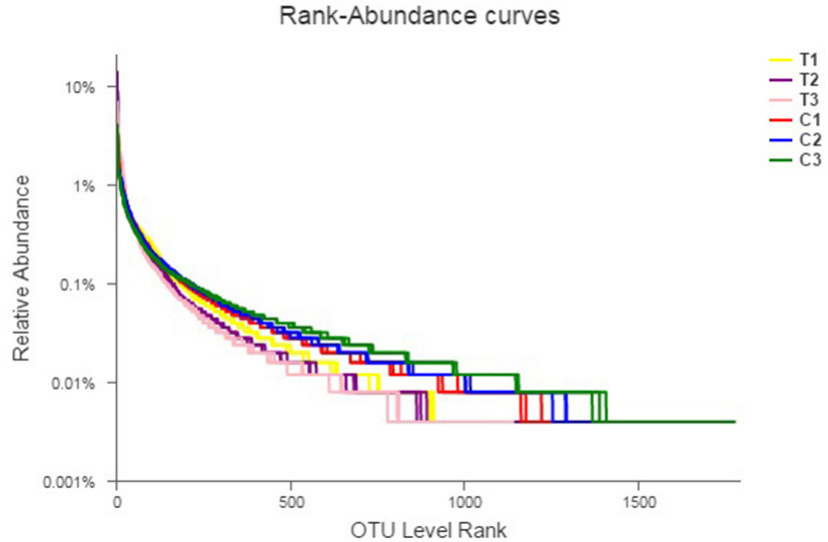
Rank-abundance curves describing the bacterial abundance and evenness. All data in the experiment represent three independent experiments.

**Figure 2:**
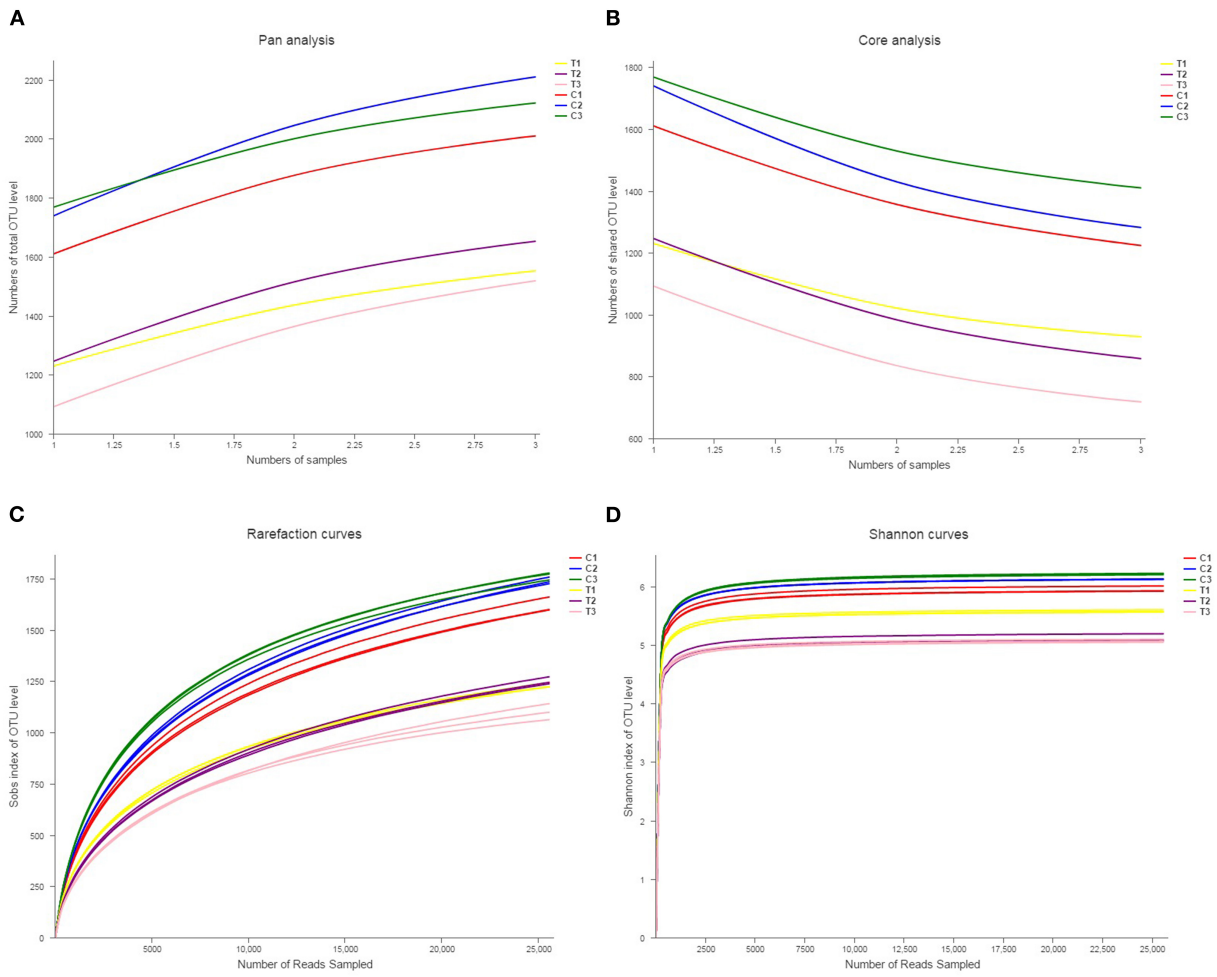
Pan-core species analysis was used to analyze total species and core species richness **(A, B)**. The rarefaction curve was drawn to quantify the soils' OTU richness **(C, D)**. All data in the experiment represent three independent experiments.

Rank abundance curves were obtained by ranking the number of species (or OTUs) at a certain taxonomic level as the horizontal coordinate and the relative percentages of the number of species at that taxonomic level as the vertical coordinates. Low-quality reads were removed from the OTUs, and investigation was conducted using rank abundance curves for the analysis of bacterial abundance and evenness (Figure 1). The curve width represented bacterial abundance, and the curve shape indicated the evenness of soil bacteria. Changes in total species and core species richness were described through Pan–Core species analysis (Figures 2A, B).

Alpha (α) diversity measurements were calculated to describe microbial diversity, such as OTU Sobs, Chao, Ace, Shannon, Simpson's, and coverage diversity estimators (Table 3). Initially, the coverage diversity indicator of each sample was over 98%, thereby indicating that the identified 16s rDNA sequences can represent the majority of bacteria present in the samples. Similarly, the uncontaminated samples had the highest Sobs, Shao, Ace, and Shannon diversity estimators but had the lowest Simpson's diversity estimator in all depths compared with polluted samples. Table 3 shows that the bacterial microbiomes ranged from 1,062 to 1,777 in the Sobs diversity estimator, 1,253.3 to 2,190.3 in the Chao diversity estimator, 5.04 to 6.22 Shannon diversity estimator, and 0.00501 to 0.0288 in the Simpson diversity estimator.

**Table 3:** Alpha diversity analysis for microbial diversity.

	**Control soils**			**Chromium soils**		
	**0–20 cm**	**20–40 cm**	**40–60 cm**	**0–20 cm**	**20–40 cm**	**40–60 cm**
Ace	2,030.431 ± 9.7192	2,185.8218 ± 33.5308	2,053.4268 ± 30.1319	1,579.0002 ± 3.8575*	1,683.8215 ± 9.8606*	1,430.823 ± 179.3792*
Chao	2,045.4253 ± 15.8363	2,199.5798 ± 28.9574	2,090.3491 ± 34.3777	1,595.0699 ± 12.2678*	1,677.997 ± 5.1928*	1,446.1553 ± 173.7856*
Coverage	0.9859 ± 0.0029	0.9847 ± 0.0021	0.9868 ± 0.0009	0.9915 ± 0.0011*	0.9879 ± 0.002*	0.992 ± 0.0002
Shannon	5.9618 ± 0.0403	6.1274 ± 0.0019	6.2207 ± 0.0132	5.5873 ± 0.0251*	5.1206 ± 0.0594*	5.0834 ± 0.0191*
Simpson	0.0069 ± 0.0005	0.0052 ± 0.0001	0.0058 ± 0.0002	0.0098 ± 0.0004*	0.0264 ± 0.0029*	0.0169 ± 0.0004*
Sobs	1,688.6667 ± 30.0888	1,807 ± 56.0268	1,798.3333 ± 35.1331	1,331.3333 ± 26.0256*	1,331 ± 31.3209*	1,202.6667 ± 112.3848*

Data of diverse depths between control and chromium soil groups were represented as mean ± SD. Values in the same row with “*” were significantly different (*p* < 0.05) in the same depth between control and chromium groups, respectively. All data in the experiment were performed for triple independent experiments.

Rarefaction curve analysis was used in comparing the OTU indicators at different sequencing depths and taxon richness, and whether the acquired sequence amounts were sufficiently quantified was determined. The rarefaction curves in Figures 2C, D showed similar patterns for all soil samples, showing that soils were equally sampled and sufficiently sequenced.

### 3.3. Bacterial community composition complexity

**Figure 3:**
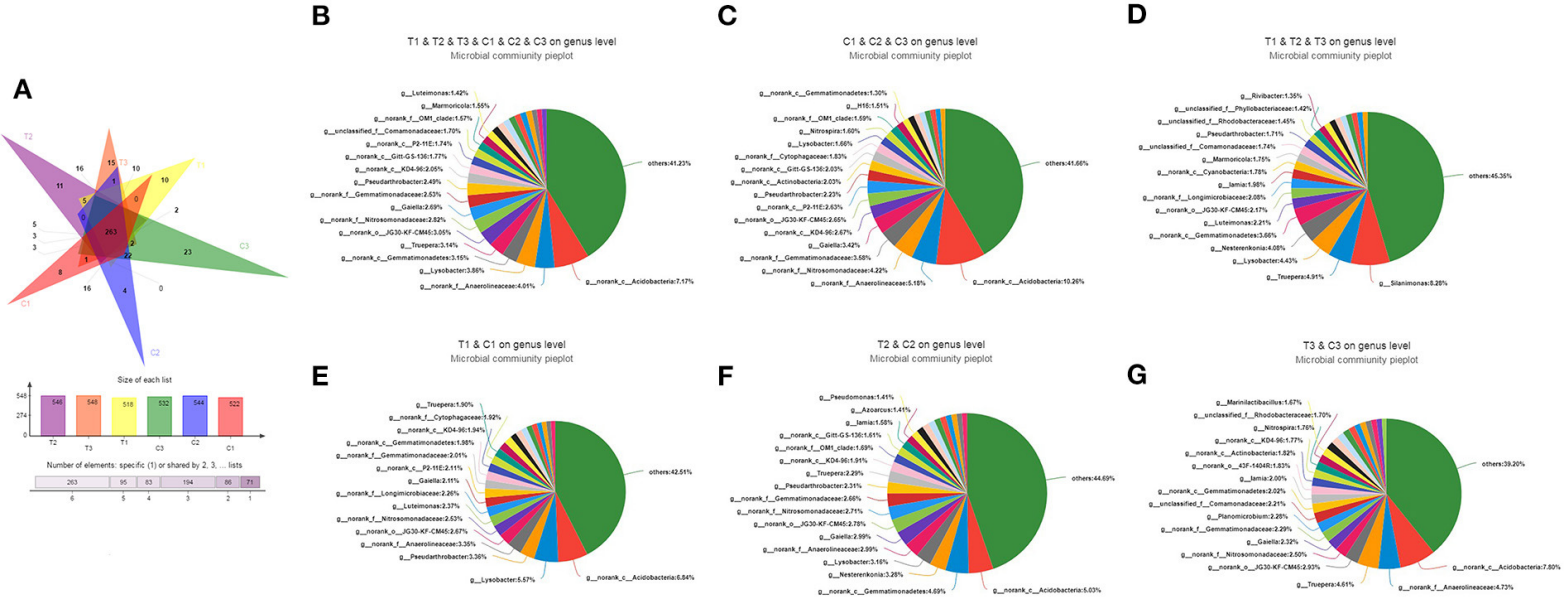
Venn diagram evaluating the distribution of OTUs among the different treatments to reflect the differences and similarities of soil samples **(A)**. Pie diagrams were used to evaluate the distribution of genus among the C1, C2, C3, T1, T2, and T3 **(B)**, C1, C2, and C3 **(C)**, T1, T2, and T3 **(D)**, C1 and T1 **(E)**, C2 and T2 **(F)**, and C3 and T3 **(G)**, respectively. All data in the experiment represent three independent experiments.

As shown in Figure 3A, the Venn diagram used to evaluate out distribution in different treatments at the genus level reflected that 263 OTUs were common in all soil samples. In addition, up to 400 and 433 OTUs belong to uncontaminated and polluted soils, respectively. C1 and T1 had 366 OTUs, C2 and T2 had 377, and C3 and T3 had 367. However, C1, C2, C3, T1, T2, and T3 had 8, 4, 23, 10, 11, and 15 unique OTUs, respectively. At the genus levels, *Acidobacteria* was the dominant bacterium in all soil groups (C1–C3 and T1–T3, 8.06%, Figure 3B), control groups (C1–C3, 10.26%, Figure 3C), C1 and T1 groups (6.84%, Figure 3E), C2 and T2 groups (5.03%, Figure 3F), and C3 and T3 groups (7.80%, Figure 3G). However, *Silanimonas* was the dominant bacterium in the polluted soil groups (T1–T3, 8.28%, Figure 3D).

**Figure 4:**
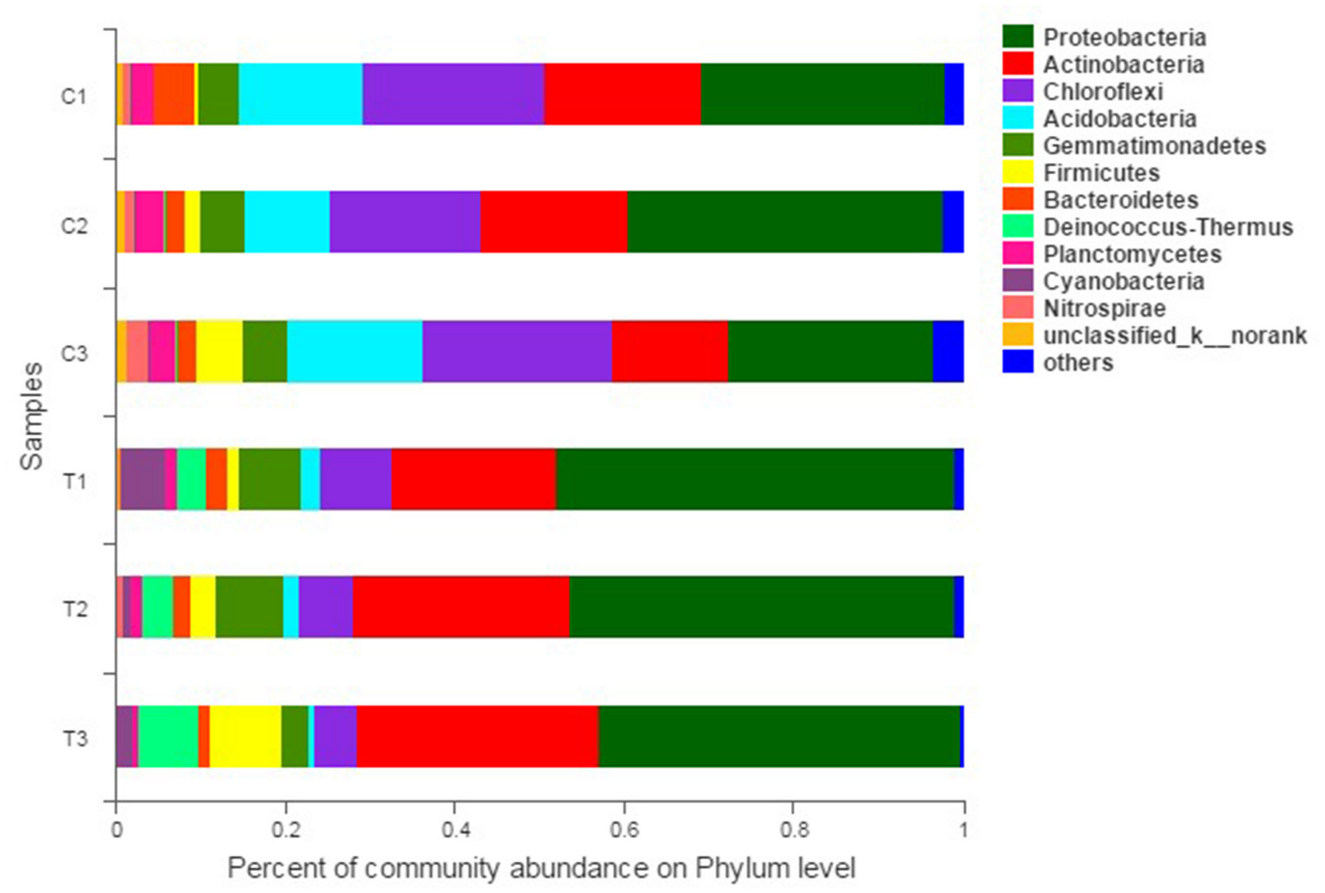
Bacterial community compositions and structures of soil samples. Bar diagrams were made to present the relative abundance levels at the phylum level of all samples. All data in the experiment represent three independent experiments.

**Figure 5:**
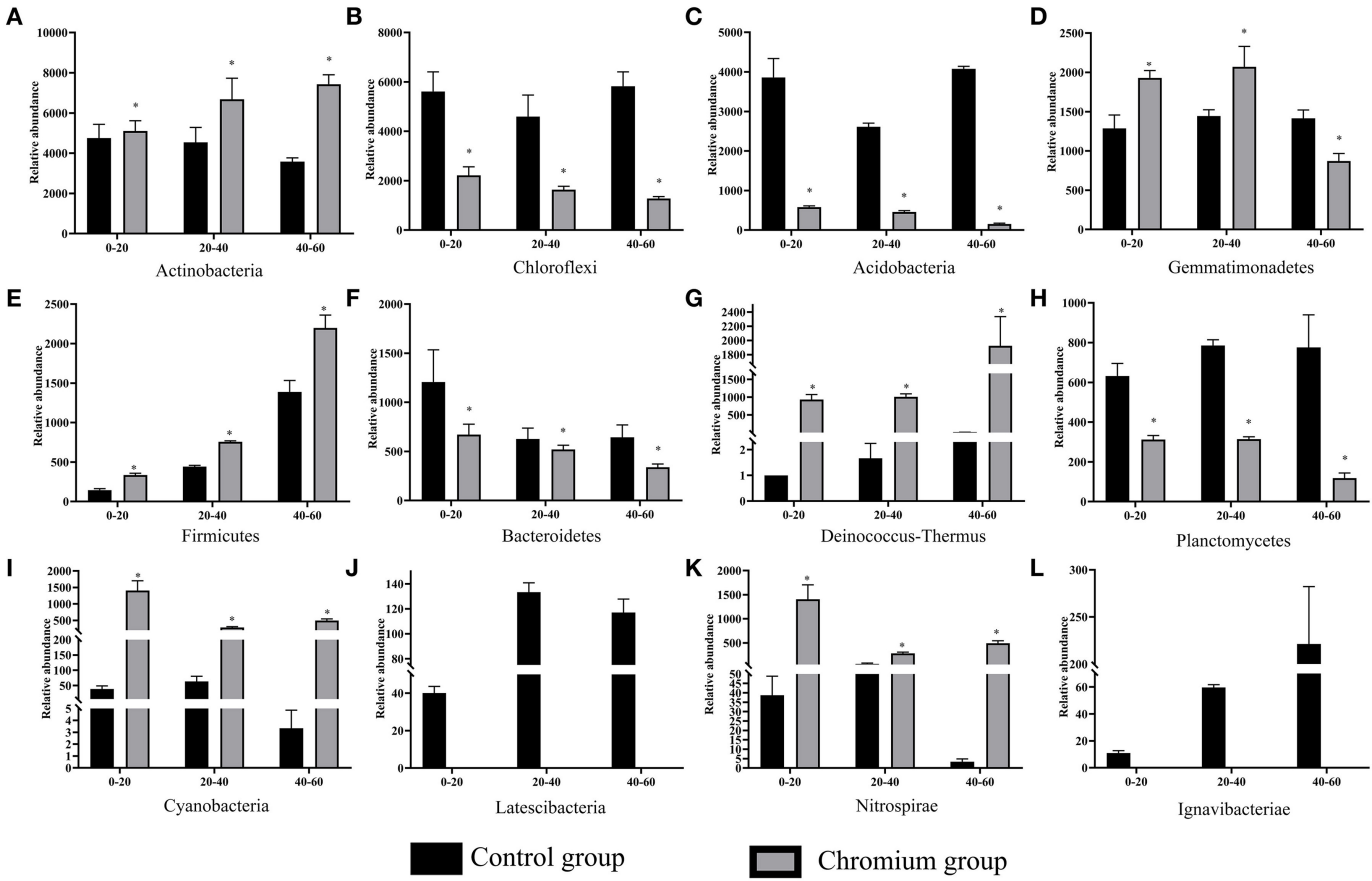
Significant differences in bacterial community composition **(A–L)** were analyzed at the phylum level. Values in the same row with “*” were significantly different (*p* < 0.05) at the same depth between the control and chromium groups, respectively. All data in the experiment represent three independent experiments.

**Figure 6:**
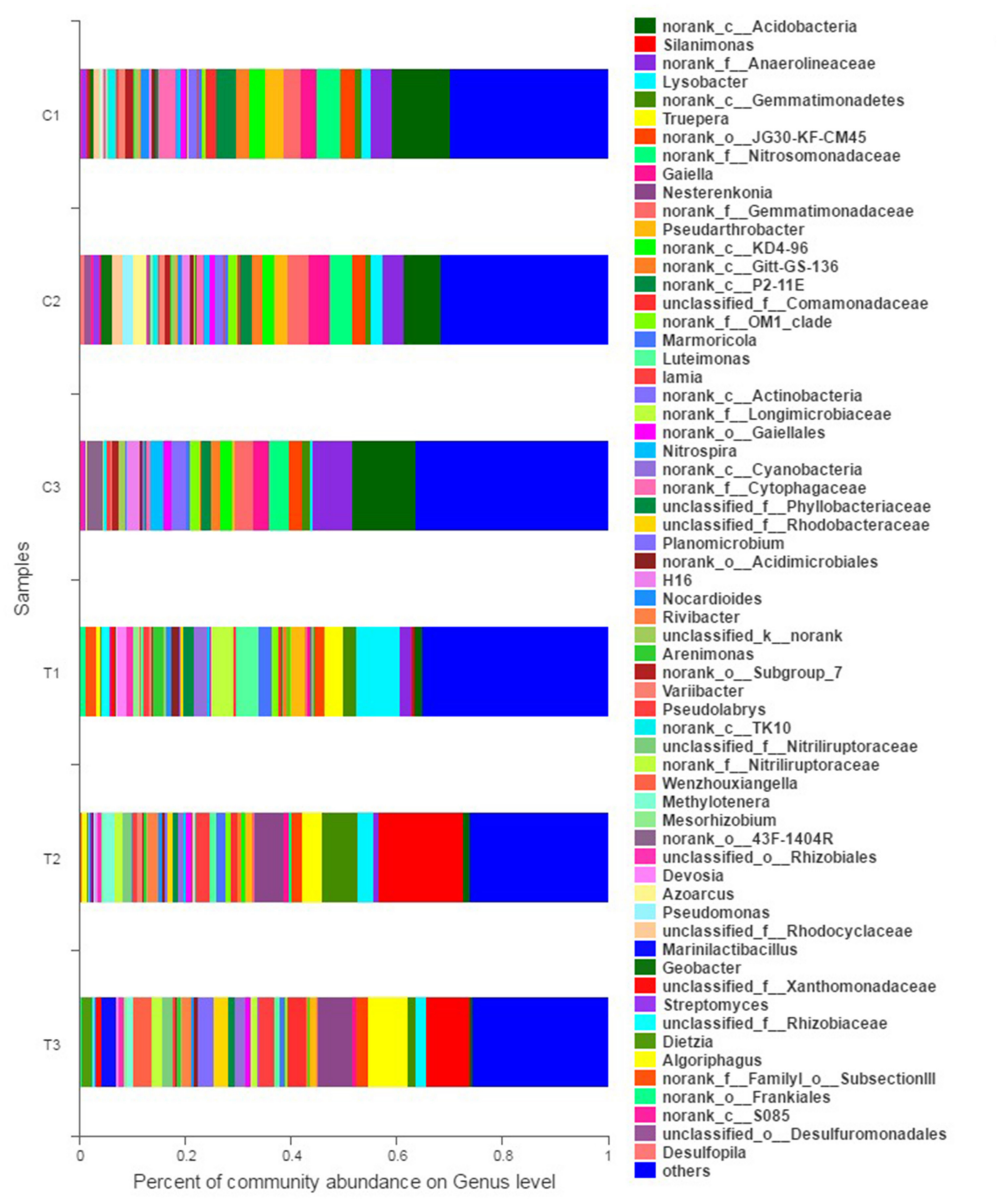
Bacterial community analysis was performed to present the relative abundance at the genus level. All data in the experiment represent three independent experiments.

The composition and structure of bacterial communities in the samples were analyzed at different taxonomic levels. Uncontaminated and contaminated groups showed analogous bacterial diversity but differed in terms of abundance to a certain extent (Figures 4, 6). Bacterial community analysis revealed relative abundance at the phylum level (Figure 4). Proteobacteria was the predominant phylum, and Actinobacteria was the secondary phylum. The relative abundance of bacterial phyla changed in the two soil types. In the C1 samples, Proteobacteria was 28.95% of the total bacteria, and the percentages of *Chloroflexi* in C1, C2, C3, T1, T2, and T3 were 21.55, 17.78, 22.24, 8.50, 6.37, and 4.97%, respectively. *Acidobacteria* and *Chloroflexi* decreased, whereas *Proteobacteria* and *Deinococcus-*Thermus were enriched in the Cr(VI)-polluted soils. At the phylum level, significance analysis of the relative abundance indicated that the significance levels of *Actinobacteria, Gemmatimonadetes, Firmicutes, Deinococcus*-*Thermus, Cyanobacteria*, and *Nitrospirae* in the chromium groups were higher than those in the control groups, whereas the significance levels of *Chloroflexi, Acidobacteria, Bacteroidetes*, and *Planctomycetes* were lower than those of the control groups. Additionally, *Latescibacteria* and *Ignavibacteriae* were unique in the control groups (Figure 5).

The abundance of relative bacterial community at the genus level is illustrated in Figure 6. *Norank Acidobacteria, norank Anaerolineaceae*, and *norank Nitrosomonadaceae* were the dominant genera in the control groups (C1–C3). However, the dominant genera in polluted soils were diverse when the contaminants were input. Specifically, *Lysobacter* (8.33%), *Luteimonast* (4.36%), and *norank Longimicrobiaceae* (4.04%) were dominant in T1; *Silanimonas* (15.94%), *norank Gemmatimonadetes* (6.71%), and *Nesterenkonia* (5.45%) were dominant in T2; and *Silanimonas* (8.27%), *Truepera* (7.28%), and *Nesterenkonia* (6.52%) were dominant in T3.

**Figure 7:**
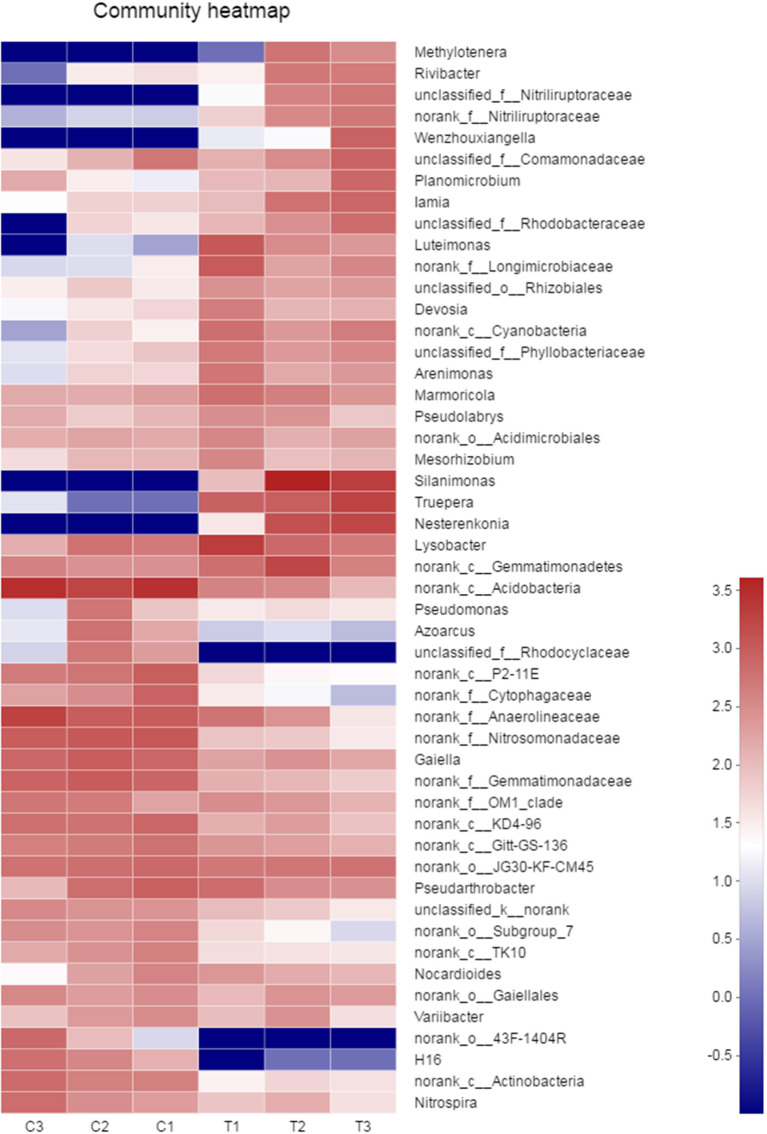
The similarity and differences in bacterial community structures in the soil samples were analyzed by heatmap. All data in the experiment represent three independent experiments.

A heatmap of microbial communities was further analyzed for the identification of the similarity and differences among bacterial community structures in the soil samples. The genera of the control groups (C1–C3) had a cluster pattern of bacterial community composition different from the pattern observed in polluted soils (T1–T3; Figure 7). At the genus level, the relative abundance of *Methylotenera, Rivibacter, Nitriliruptoraceae, Wenzhouxiangella, Luteimonas, Silanimonas, Truepera*, and *Nesterenkonia* in the chromium groups were higher than those in the control groups, whereas the relative abundance of *Rhodocyclaceae, 43F-1404R*, and *H16* were lower than those in the control groups.

**Figure 8:**
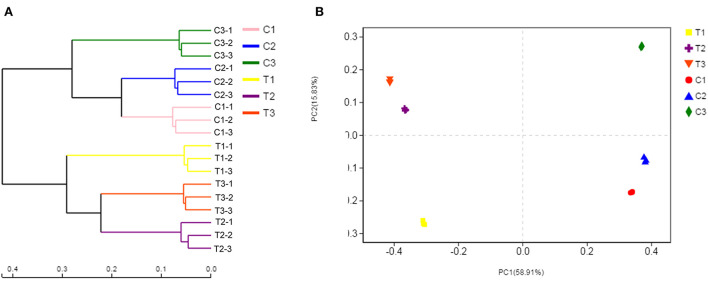
Soil samples were separated by hierarchical clustering analysis and PCoA. The similarity and differences in the bacterial community structures were determined by hierarchical clustering tree analysis **(A)**. PCoA analysis of the bacterial communities at the OTU level **(B)**. All data in the experiment represent three independent experiments.

Bacterial community composition varied by chromium content and soil depth. Therefore, the samples were separated by principal coordinate analysis (PCoA) and hierarchical clustering analysis, as illustrated in Figure 8B. At the OTU level, the PCoA indicated the clear separation of the bacterial communities in different groups. The microbiota of the T1, T2, and T3 samples was distinct from that of the C1, C2, and C3 samples. The first axis explained the 58.91% cumulative percentage variance of species, and the second axis explained 15.83%. The two axes explained a total of 74.74% variance of species. Meanwhile, further analysis of microbial communities was executed through a hierarchical clustering tree, which was used in identifying similarities and differences among bacterial community structures (Figure 8A). The microbial community structures in uncontaminated and polluted soils substantially differed, and these structures were divided into two clusters.

**Figure 9:**
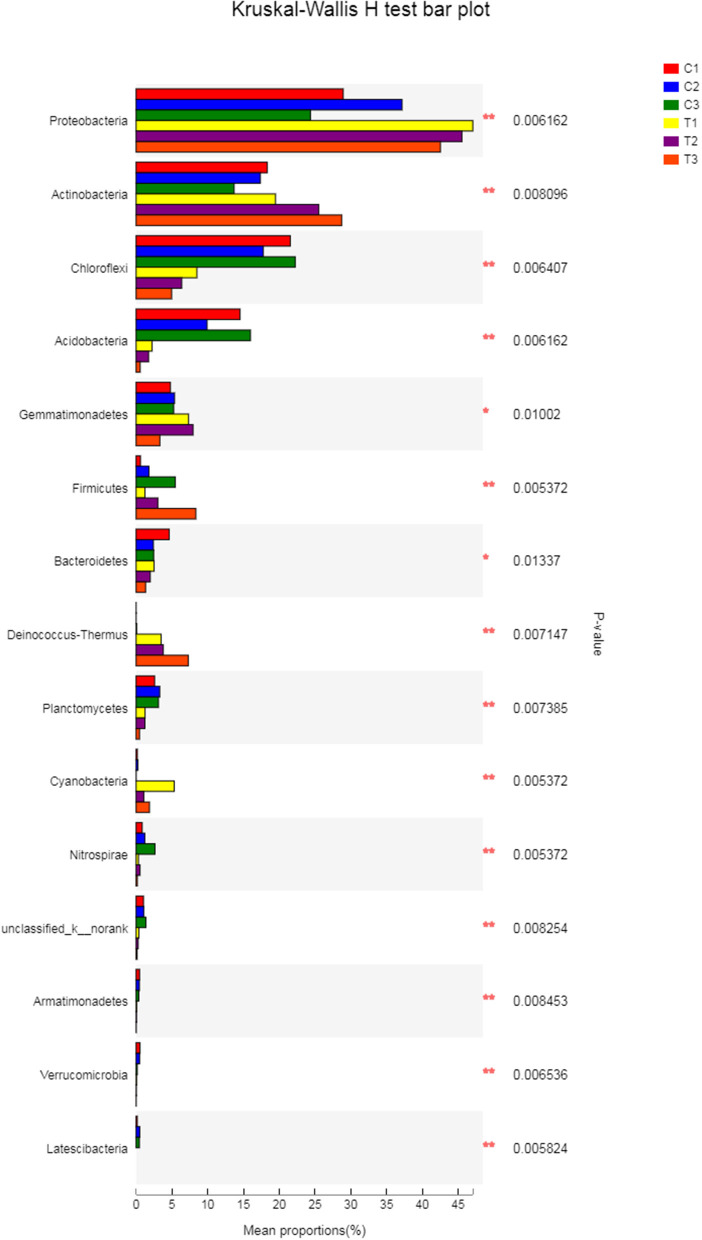
The Kruskal-Wallis *H*-test was chosen to detect the richness of microbial communities exhibited by different samples at the phylum level. All data in the experiment represent three independent experiments.

**Figure 10:**
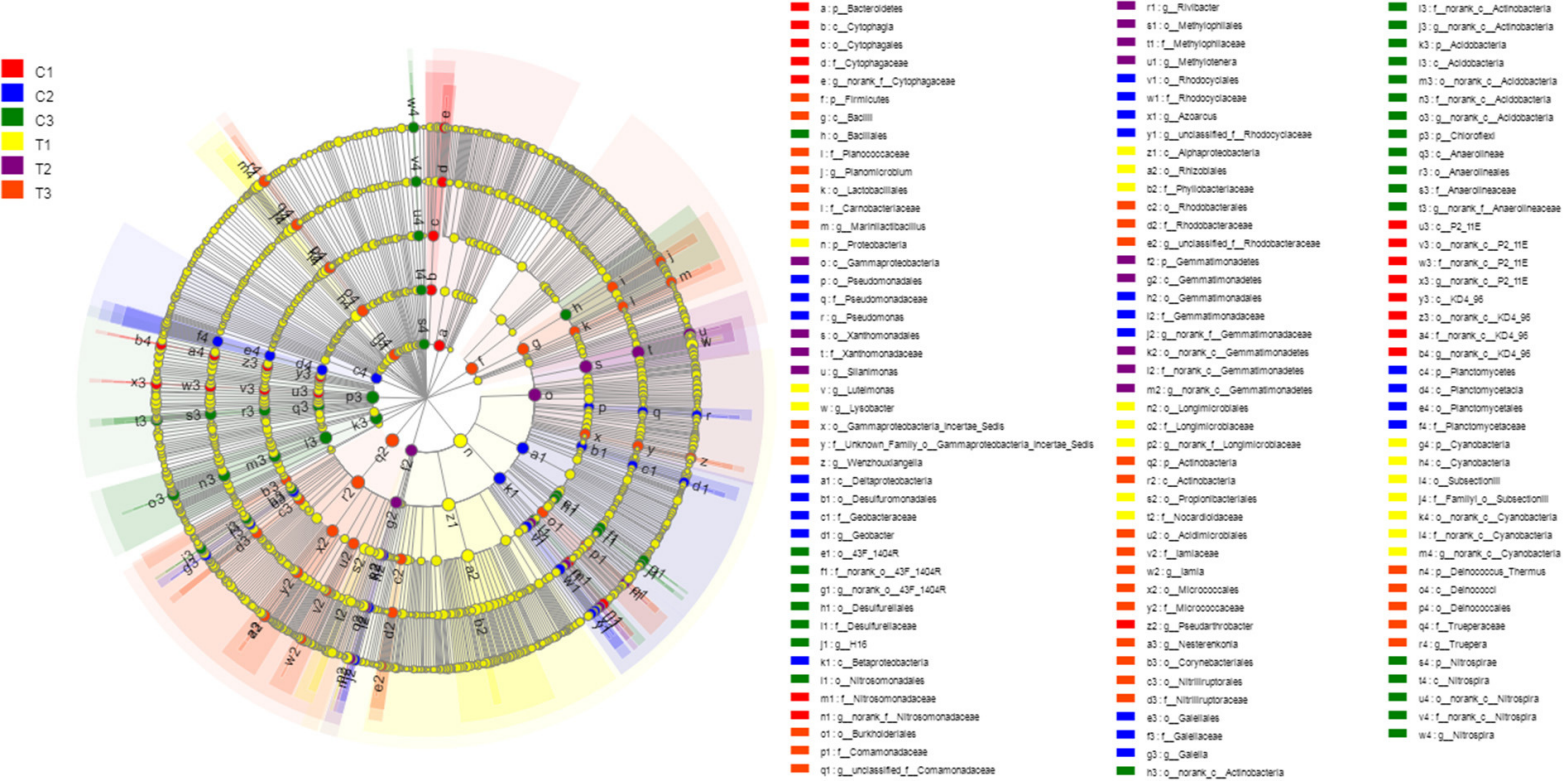
LEfSe was used to discriminate the microbial communities of all soil samples. Significantly affected microbial taxa were shown in LEfSe multi-level species hierarchy tree diagram. All data in the experiment represent three independent experiments.

**Figure 11:**
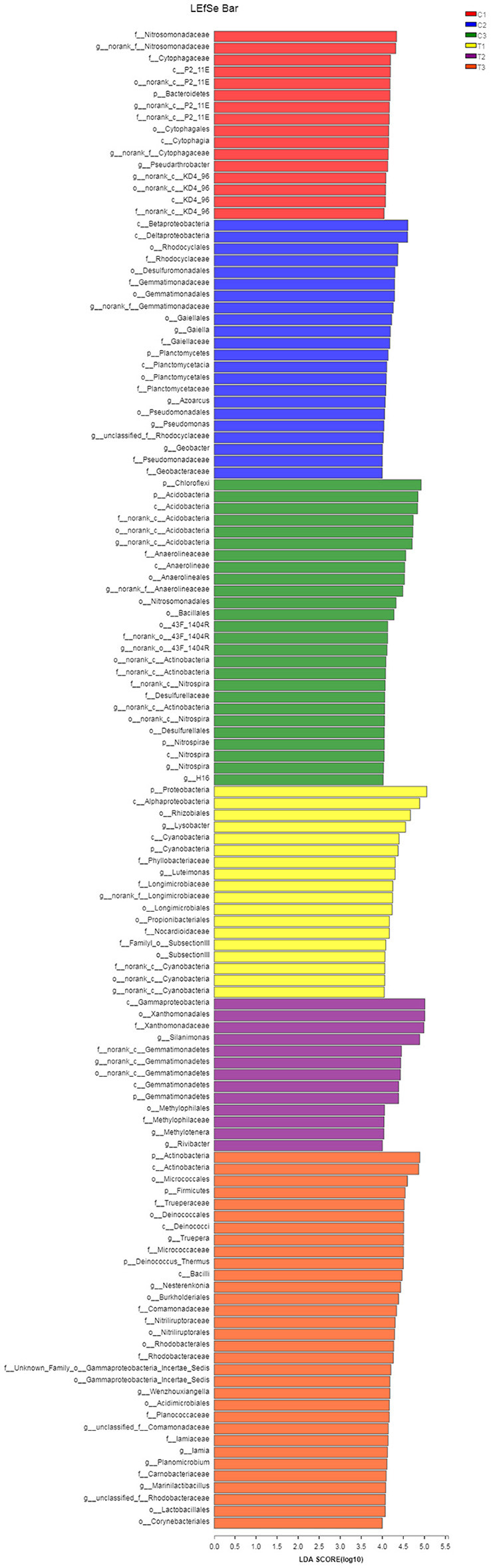
LEfSe was used to discriminate the microbial communities of all soil samples. LDA discriminant bar charts were used to count microbial taxa that differed significantly in all groups. All data in the experiment represent three independent experiments.

A Kruskal-Wallis *H*-test was used for detecting richness exhibited by different samples of microbial communities at the phylum level. *Proteobacteria, Actinobacteria, Chloroflexi*, and *Acidobacteria* were the dominant bacterial phyla in all samples, but the C1–C3 and T1–T3 samples differed in the abundance of these bacteria (Figure 9). In addition, LDA effect size analysis (LEfSe) was used to discriminate between microbial communities in uncontaminated soils and those in polluted soils. Figures 10, 11 show differences in bacterial taxa. The dominant bacterial phyla were *Bacteroidetes, Verrucomicrobia, Armatimonadetes, Chlorobi*, and *WS2* in C1; *Planctomycetes* and *Latescibacteria* in C2; *Chloroflexi, Acidobacteria*, and *Nitrospirae* in C3; *Proteobacteria, Cyanobacteria*, and *BRC1* in T1; *Gemmatimonadetes*, BJ_169, *M6*__*Dependentiae*, and *Parcubacteria* in T2; and *Actinobacteria, Deinococcus*_*Thermus*, and *Firmicutes* in T3.

### 3.4. Correlation among environmental factors, soil samples, and bacterial floras

**Figure 12:**
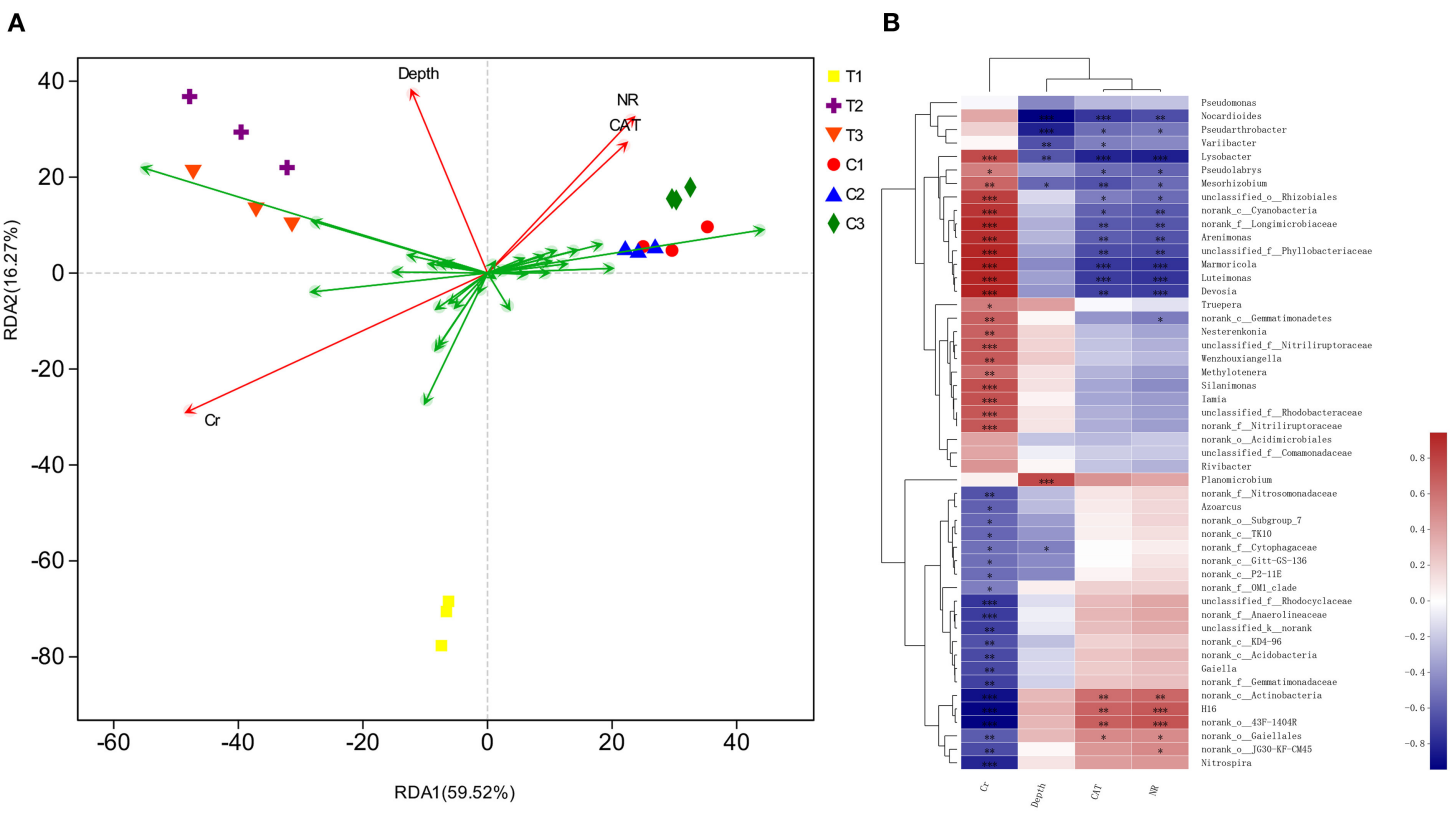
Based on different environmental factors, RDA result of bacterial communities **(A)** and heatmap of correlations **(B)** in soil samples. Values in the same row with “*, **, and ***” were significantly different (*p* < 0.05, *p* ≤ 0.01 or *p* ≤ 0.01). All data in the experiment are from three independent experiments.

The correlation of environmental factors, soil samples, and bacterial floras were further assessed by redundancy analysis (RDA) (Figure 12). RDA results illustrated that the sequence of contributors to the diversification was as follows: bacterial community's chromium content > NR > depth > CAT (*P* < 0.05). The factors involved in chromium content, soil depths, and enzyme levels explained 85.79% diversity of the bacterial communities. The RDA1 axis explained 59.52% of the total variance separating C1–C3 groups from T1–T3 groups.

### 3.5. Changes in soil enzymes

**Figure 13:**
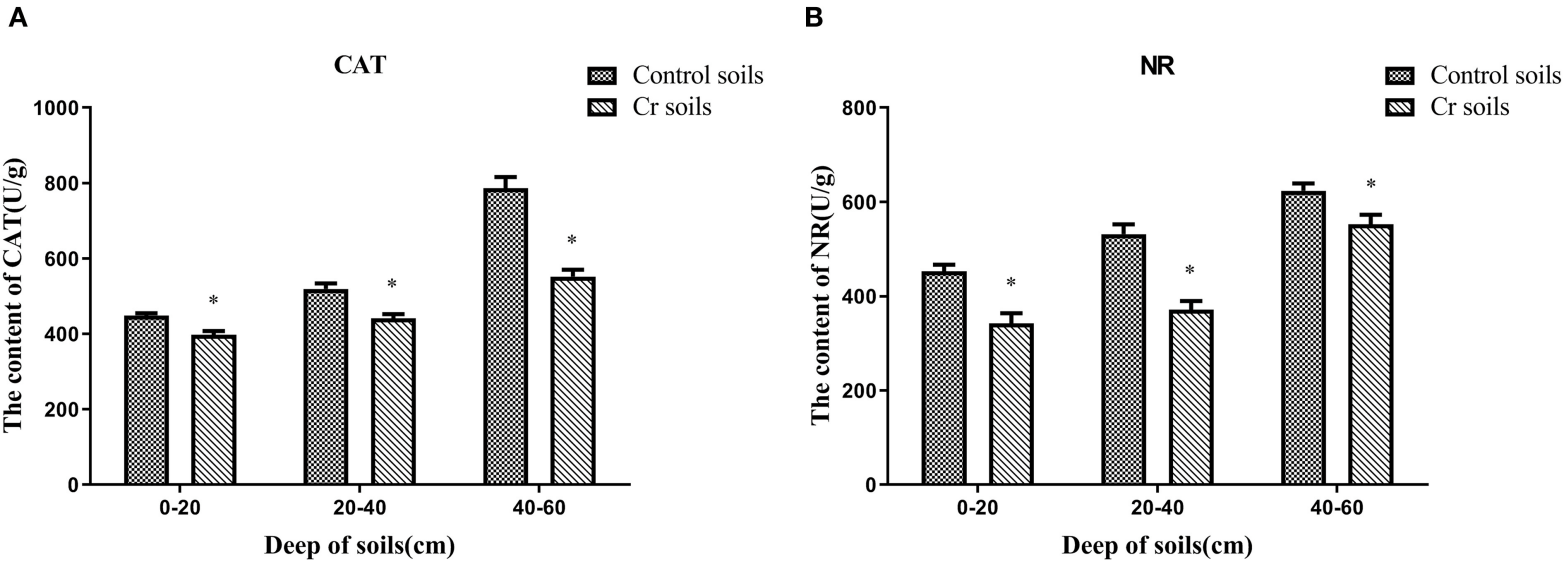
Antioxidant enzyme (NR and CAT) contents of soils **(A, B)**. Data showing the diverse depths between Control and Chromium soil groups are presented as the mean ± SD. Values in the same row with “*” were significantly different (*p* < 0.05) in the same depth between the Control and Chromium groups. All data in the experiment represent three independent experiments.

The content of antioxidant enzymes (NR and CAT) decreased in Cr(VI)-polluted soils at all depths compared with those in C1–C3. Moreover, the NR and CAT content increased with depth (Figure 13).

## 4. Discussion

According to the national survey bulletin on soil pollution in China, 34.9% of all wastelands surveyed exceeded the standard in terms of the amount of pollutants. The involved pollutants were zinc, chromium, mercury, lead, arsenic, and polycyclic aromatic hydrocarbons, which were produced by chemical and metal manufacturing industries (22). In the present study, chromium content in T1–T3 notably exceeded the highest national control standard value of 1,300 mg/kg (GB 15618-2018). The high risk of potentially toxic metals migrating to the environment can lead to the frequent migration of potentially toxic metals into the soil (23).

As a result of the influences of soil microbes and soil environments, differences in soil microbes are inconspicuous even after long-term succession (24, 25). Hence, bacterial microbiome communities were used in identifying changes in Cr(VI)-polluted soils.

In this study, T1–T3 curves were steeper than C1–C3 curves, indicating that the species uniformity of the control groups was more consistent than T1–T3. Furthermore, OTU levels increased with depth in C1–C3, whereas those of T1–T3 were the opposite. The results obtained from the rank abundance curve highlighted a significant fraction of variations in C1–C3 and T1–T3 diversities (in horizontal and vertical axes) that can be attributed to the content, mobility, and permeability of Cr(VI). Table 2 shows that the chromium content of T1–T3 was higher than that of C1–C3 and had a decisive effect on the microbiome. Meanwhile, chromium content at different depths influenced bacterial taxa distribution and microbiome evenness.

Biodiversity is evaluated by the richness, evenness, and α diversity indices of the microbial composition (26). In the present research, Sobs, Chao, Ace, Shannon, and Simpson's diversity indices and coverage indices were assessed to represent the richness and evenness of a microbial community. The results of microbial richness in Sobs, Chao, and Ace indices were similar in different land types but varied by soil depth. The results of microbial evenness in Shannon and Simpson's diversity indices indicated an analogous phenomenon. These results revealed that soil microbial diversity was stable across soil environments; therefore, soil microbial diversity can reflect soil properties (27). Moreover, the diversity indices of the microbial composition revealed that the microbial community is crucial to the preservation of the function and stability of soil ecosystems (28–30). The results in Figures 2C, D indicate that the microbial community significantly decreased after being polluted with Cr(VI).

*Proteobacteria* and *Actinobacteria* were the dominant phyla in all soil samples, consistent with other reports (31, 32). The properties of *Proteobacteria*, a slightly acidic and metabolically diverse species, can explain the high presence of these in soil samples (33). In addition, *Actinomycetale* are common in metal-impacted soils, especially chromium-polluted soils (34–36). Analysis of the similarity tree of multiple samples indicated the similar community structures of C1–C3 and Cr(VI)-polluted soils. By contrast, the relative abundance of strains markedly differed between the control and polluted soil samples.

The findings revealed that changes in microbial diversity and abundance can affect soil microbial biomass and activity (37). Therefore, soil microbial community (diversity and structure) integrated with microbial biomass and activity might be used in developing an approach for evaluating the risk of contaminated soils. Thus, a potential method for the bioremediation of contaminated soil can involve the addition of uncontaminated soil bacteria and removal of contaminated soil bacteria for soil microbial stability. Such bacteria include *Latescibacteria* (which only existed in control soils), which can degrade organics (38).

The composition of soil bacterial communities is closely related to soil properties, such as antioxidation enzymes; hence, information regarding shifts in the antioxidation enzymes of soil may be used in predicting changes in the bacterial community after Cr(VI) pollution. In addition, soil microbial biomass and activity can sensitively assess soil quality. Pb can bind with NH2 and SH enzyme groups, thereby causing enzymes to lose their activity and preventing them from harming soil microorganisms (39, 40). Pb might underlie the mechanism for decreasing CAT and NR levels in Cr-polluted soils.

Previous reports have demonstrated that pollutants, enzyme levels, and spatial depth can reveal the diversity of soil bacterium (41, 42). Among the influencing parameters, chromium was closely related to bacterial communities' composition and structure (*r*^2^ = 0.9412, *P* = 0.001), whereas depth was weaker linked to diversity (*r*^2^ = 0.3728, *P* = 0.03). Soils were collected at three depths, but the phyla were dominated by *Proteobacteria, Actinobacteria, Chloroflexi*, and *Acidobacteria*. The depth-specific differences were not the main factors for bacterial diversity in this study.

## 5. Conclusion

MiSeq sequence results showed a significant difference between Cr(VI)-polluted soils and control soils, regardless of the depths of Cr(VI)-polluted soils. The data of soil microbial biomass indicated that the soil polluted with Cr(VI) suppressed the microbial diversity and antioxidized enzyme levels, changing bacterial community composition and structure significantly, revealing the distribution of *Latescibacteria* and *Ignavibacteriae*. The depths values and CAT levels had minor contributions to bacterial community changes. By contrast, Cr(VI) contamination was the main factor that affected the balance of bacterial microbial communities by inhibiting bacterial diversity, microbial community structure complexity, and antioxidant enzyme levels. Collectively, differences in bacterial microbiome communities and antioxidant enzyme levels of soils can facilitate the assessment of the degree of chromium contamination in soil samples. These results are critical for stabilizing the state of microbial community structures in view of the potential risk of metal accumulation in soils.

## Data availability statement

The data presented in the study are deposited in the Dryad repository, accession number is https://doi.org/10.5061/dryad.dr7sqvb2f.

## Author contributions

YL and JL: conceptualization. XW and YZ: data curation. KS: formal analysis. YL: funding acquisition. PZ: investigation. XW and HX: methodology. JL, YL, and PZ: project administration and supervision. XW and CQ: resources. HX: software. GC and YL: validation. YZ: visualization. YZ and KS: writing—original draft. YZ, YL, and JL: writing—review and editing. All authors contributed to the article and approved the submitted version.
